# Which practice characteristics are associated with the quality of cardiovascular disease prevention in European primary care?

**DOI:** 10.1186/1748-5908-8-27

**Published:** 2013-03-09

**Authors:** Sabine Ludt, Stephen M Campbell, Davorina Petek, Justine Rochon, Joachim Szecsenyi, Jan van Lieshout, Michel Wensing, Dominik Ose

**Affiliations:** 1 Department of General Practice and Health Services Research, University Hospital of Heidelberg, Voßstrasse 2, D-69115 Heidelberg, Germany; 2 University of Manchester, Health Sciences – Primary Care Group, Williamson Building, |Manchester M13 9PL UK; 3 Medical School University of Ljubljana, Department of Family medicine, Poljanski nasip 58, Ljubljana 1000, Slovenia; 4 University of Heidelberg, Institute of Meedical Biometry and Informatics, Im Neuenheimer Feld 120, Heidelberg D-69120, Germany; 5 Radboud University Nijmegen Medical Centre, Scientific Institute for Quality of Healthcare, P.O. Box 9101, Nijmegen 6500 HB The Netherlands

## Abstract

**Background:**

Prevention of cardiovascular diseases (CVD) is a major health issue worldwide. Primary care plays an important role in cardiovascular risk management (CVRM). Guidelines and quality of care measures to assess CVRM in primary care practices are available. In this study, we assessed the relationship between structural and organisational practice characteristics and the quality of care provided in individuals at high risk for developing CVD in European primary care.

**Methods:**

An observational study was conducted in 267 general practices from 9 European countries. Previously developed quality indicators were abstracted from medical records of randomly sampled patients to create a composite quality measure. Practice characteristics were collected by a practice questionnaire and face to face interviews. Data were aggregated using factor analysis to four practice scores representing structural and organisational practice features. A hierarchical multilevel analysis was performed to examine the impact of practice characteristics on quality of CVRM.

**Results:**

The final sample included 4223 individuals at high risk for developing CVD (28% female) with a mean age of 66.5 years (SD 9.1). Mean indicator achievement was 59.9% with a greater variation between practices than between countries. Predictors at the patient level (age, gender) had no influence on the outcome. At the practice level, the score ‘Preventive Services’ (13 items) was positively associated with clinical performance (*r* = 1.92; p = 0.0058). Sensitivity analyses resulted in a 5-item score (PrevServ_5) that was also positively associated with the outcome (*r* = 4.28; p < 0.0001).

**Conclusions:**

There was a positive association between the quality of CVRM in individuals at high risk for developing CVD and the availability of preventive services related to risk assessment and lifestyle management supported by information technology.

## Background

Cardiovascular diseases (CVD) are major causes of morbidity and mortality across developed countries worldwide and contribute substantially to escalating healthcare costs [1,2]. Therefore, prevention of cardiovascular disease is a priority for health care systems in most countries [3].

Patients should receive or be offered a range of appropriate services in primary care to address their acute and chronic conditions, including health promotion and targeted lifestyle advice [4]. Lifestyle advice by general practitioners to individuals takes place alongside community or population level health promotion initiatives designed to improve the overall health of populations [5].

However, the delivery of preventive services is often a low priority in family practices [6]. It has been stated that practice systems and processes are not optimally designed to address the care of individuals with preventive needs, for instance the delivery of behavioural counselling [7,8]. Despite various approaches to improve structure and organisation of services delivered in primary care [9,10] little is known about the relationship between structural and organisational practice characteristics and the quality of care [11], especially as it relates to preventive care. It has been shown that some practice capabilities such as the use of electronic health records were associated with improved clinical performance [12]. The National Committee for Quality Assurance (NCQA) defined structural elements in practices with a focus on information technology to quality practices as medical homes according to the patient-centred medical home model [13]. However, key relationships between structural capabilities of practices and their performance on measures of clinical quality in routine primary care settings have not been clearly detected [12,13].

The aim of this study was to determine the effect of structural and organisational practice characteristics on the quality of preventive care for patients at high risk for developing CVD in routine primary care within different European health care systems.

## Methods

This study was part of the European Practice assessment (EPA) - Cardio project (2006–2010), which focused on the assessment of cardiovascular risk management in European primary care in populations at different risk levels. In the first stage of the project we developed quality indicators to measure cardiovascular prevention and care [14] and identified additional measures that were piloted before being used in this study [15].

### Samples

**Figure 1:**
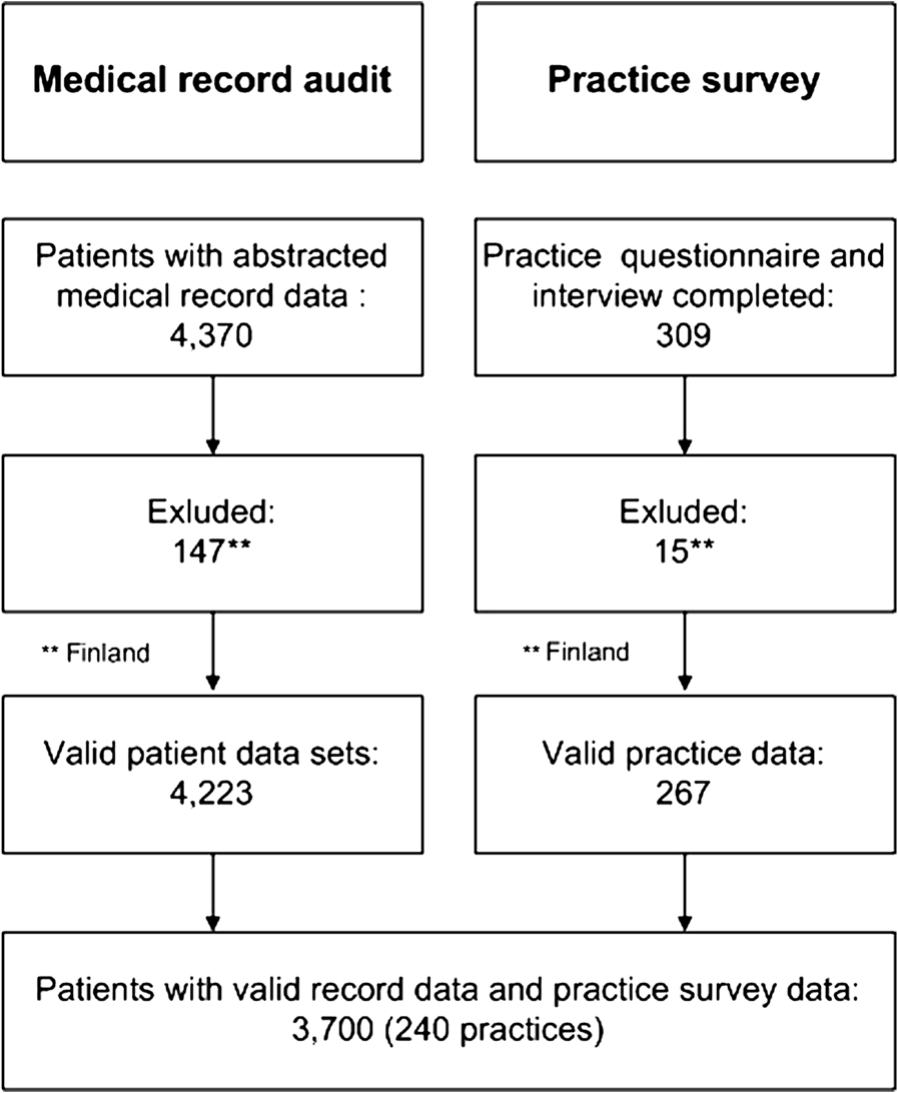
Data flowchart.

This cross-sectional observational study was conducted between 2008 and 2009 and involved ten European countries: Austria, Belgium, Finland, France, Germany, the Netherlands, Slovenia, Spain, Switzerland and the United Kingdom (UK). In this part of the study, Finland was excluded due to insufficient practice data (Figure 1). The study design and data collection methods have been published in detail elsewhere [15,16]. In order to achieve a predefined degree of statistical accuracy a representative sample of 36 general practices per country was aimed for. Each practice had to identify patients at high risk for CVD from the practice register according to the criteria listed in Table 1. Of the eligible patients, a sample of 30 patients per practice was randomly selected to be posted a questionnaire and informed written consent form, with the aim of recruiting at least 15 patients per practice. The study was approved by relevant ethics committees in all participating countries.

**Table 1:** Inclusion and exclusion criteria

**Inclusion criteria**	**Exclusion criteria**
- High- risk defined by risk calculation with recommended tools according to national guidelines, e.g. > 20% CVD event risk as calculated by Framingham	1. Patients with established CVD (including ischemic heart disease, myocardial infarction, angina pectoris, coronary surgery or revascularisation procedures, ischaemic stroke, transient ischemic attack, claudication or peripheral vascular disease)
**or**	2. Patients with diabetes
- Patients with 3 out of the following 4 risk factors: hypertension, hypercholesterolemia, smoking, men over 60 years of age	3. Terminal illness, cognitive disorders (e.g. dementia), psychiatric diseases (e.g. schizophrenia) and lack of language knowledge

### Measures

The main outcome measure was the quality of cardiovascular risk management (CVRM) that was measured against eleven quality indicators developed in the earlier stage of the EPA-Cardio project [14]. These indicators were related to guideline recommendations such as the recording of clinical and behavioural risk factors, control of clinical risk factors and behavioural advice. Data were abstracted from medical records using a previously developed paper-based abstraction form. A composite quality score was created by summing up the number of indicators achieved (9 process and two outcome indicators) divided by the number of applied indicators expressed as percentages.

Data on practice characteristics and systems were collected by posting questionnaires to the practice team and by face-to-face or telephone interviews with the leading General Practitioners (GPs) in each practice. The questionnaire contained questions to characterize the practice according to size, location, and number and function of practice staff. The instrument included also questions reflecting quality indicators on practice management and organisation that were partly developed during the EPA-Cardio project [14] and partly derived from the EPA practice-management instrument [17]. The practice items represented organisational processes specific to CVD-prevention and care (33 items) and more generic practice management features in the three dimensions ‘information process and technology’ (11 items), ‘organisation of chronic care and prevention’ (19 items) and ‘quality improvement’ (13 items). The interview guide contained questions concerning preventive CVD care, e.g. concerning risk assessment, lifestyle counselling and cardiovascular quality improvement activities and projects.

To create practice scores, we aggregated these items using a factor analysis method for binary variables (CATPCA) [18]. During this analysis, we identified 27 binary items that were not correlated with each other and had factor loadings over 0.4 in four dimensions: ‘preventive services’ (PrevServ - 13 items), ‘electronic medical record’ (EMR - 5 items), ‘chronic care management’ (CCM - 6 items) and ‘quality management’ (QM - 3 items). The explained variance was 54%. Scores were created by summing up the number of ‘yes’- answers and included in the multilevel analysis and were descriptively presented as percentages of all included items in each score.

### Analyses

We analysed the association between organisational practice characteristics and the quality of CVRM measured by a composite performance score, ranging between zero and eleven (or 0–100%) with higher scores indicating better quality of CVRM in patients at high risk of developing CVD. Due to the hierarchical structure of the data, linear multilevel regression analysis was applied, which takes into account the non-independence of patient observations (level 1) nested within practices (level 2) and these nested within countries (level 3). Several models were evaluated starting with an intercept-only (null) model (M0) without any predictor variables. Variance partition coefficients in each level were calculated using the restricted maximum likelihood (REML) method. This model was used as a reference for comparing the size of contextual (practice or country) variations of outcomes in subsequent models. The random-part results of the null model (M0) are reported together with the corresponding intra-class correlations (ICC) at the practice and country level. The differences between the null model and the following models show to which extent the explanatory variables explain the observed variation in the outcome.

The next model (M1) included only the patient-level predictors as fixed effects. In contrast, the next two models included only the practice-level predictors as fixed effects: M2.1 included only the four practice scores from CATPCA, M2.2 additionally contained the characteristics related to practice location and size. The final model (M3) included a total set of eight potential explanatory variables, two at the patient level and six at practice level. Explanatory variables on country level were not examined. As only one of the four practice scores showed a significant association with the outcome, we separated the 13 single items of this score and calculated the association with the outcome for each item. By this step we identified 5 items, calculated a score of these 5 variables and included this score together with the variables at the patient level, practice size and location in a sensitivity analysis (M4).

Only patients for whose data on all explanatory variables on the different levels were available could be included in the multilevel analysis. A non-responder analysis was performed between those patients included in the final dataset and those not included because of missing data. The significance level was set to 5% (two-sided). Regression coefficients (*r*) and corresponding two-sided 95% confidence intervals (CI) were calculated and considered statistically significant if the CI excluded zero. Statistical analyses including CATPCA were carried out by using SPSS version 20.0 (SPSS Inc., Chicago, IL, USA). The multilevel analyses were conducted using the procedure PROC MIXED in SAS version 9.2 (SAS Institute, Cary, NC).

## Results

### Patient and practice characteristics

A total number of 267 practices and 4,223 patients at high risk for developing CVD participated in the study. The mean age of the participants was 66.6 years (SD 9.1) years and 27.5% were female. The number of participating practices per country ranged from 22 (Switzerland) to 36 (Slovenia, Spain, the Netherlands and the UK). 31.2% of the practices were located in large towns (i.e. more than 100,000 inhabitants) and 47.5% had two or more GP full time equivalents (FTE). The overall achievement was 53.0 for the practice score ‘Preventive Services (PrevServ)’, 81.1% for ‘Electronic Medical Record (EMR)’, 53.3% for ‘Chronic Care Management (CCM)’ and 41.8% for ‘Quality Management (QM)’ (Table 2). The percentages of the single-item achievements of these scores are indicated in Table 3. Scores varied between countries with maximal scores in the UK and minimal scores in France and Switzerland (Table 2).

**Table 2:** Practice characteristics and organisational measures

**Countries**	**Practices**	**Patients**	**Urban**^**1**^	**FTE**^**2 **^**GP ≥ 2**	**Practice scores**							
					**PrevServ**^**3**^		**EMR**^**4**^		**CCM**^**5**^		**QM**^**6**^	
	**N**	**N**	**%**	**%**	**%**	**SD**^**7**^	**%**	**SD**	**%**	**SD**	**%**	**SD**
Austria	24	285	25.0	0.0	22.1*↓	16.4	73.3	23.3	42.4	29.5	47.2	31.0
Belgium	24	266	9.1	50.0	35.6*↓	19.0	85.8	30.3	20.1*↓	20.3	30.6	40.4
France	29	377	21.4	50,0	24.1*↓	17.2	86.2	18.6	21.8*↓	31.2	23.0^+^↓	29.7
Germany	26	470	16.7	22.2	28.1*↓	21.1	70.8	41.3	56.4	35.6	24.4^+^↓	27.6
Netherlands	34	467	35.3	23.5	47.1	19.7	95.3*↑	11.1	71.1*↑	19.8	23.5*↓	25.3
Slovenia	36	842	25.0	51.6	76.1*↑	13.6	54.4*↓	30.8	41.7^+^↓	25.4	30.6	36.8
Spain	36	652	41.7	97.2	89.5*↑	9.4	99.4*↑	3.3	88.0*↑	12.4	64.8*↑	22.5
Switzerland	22	329	0.0	5.9	28.7*↓	15.8	50.9*↓	30.7	9.8*↑	16.8	10.6*↑	26.0
UK	36	535	76.5	77.8	87.2*↑	8.4	100.0*↑	0.0	94.4*↑	9.8	98.1*↑	7.7
Total	267	4223	31.2	47.5	53.0	31.1	81.1	29.2	53.6	36.7	41.8	38.7

^1^ Urban = Practice in towns with more than 100.000 inhabitants (more than 30.000 for Slovenia).

^2^ FTE = Full time equivalent.

^3^ PrevServ = Preventive Services (score: % of ‘yes answers’ from 13 items) (Table 3).

^4^ EMR = Electronic medical record (score: % of ‘yes answers’ from 5 items) (Table 3).

^5^ CCM = Chronic Care Management (score: % of ‘yes answers’ from 6 items) (Table 3).

^6^ QM = Quality Management (score:% of ‘yes answers’ from 3 items) (Table 3).

^7^ SD = Standard Deviation

* Scores significantly deviating from the mean of all countries (p < 0.0001).

^+^ Score significantly deviating from the mean of all countries (p = <0.01).

↑ Country score higher than the mean score of all countries.

↓ Country score lower than the mean score of all countries.

**Table 3:** Structural and organisational characteristics of practices (N = 267)

**No**	**Questionnaire items**	**Yes answers%**	**Factor loads**
**Preventive services (PrevServ) - 13 Items**			
1	Is the CVD risk assessment tool integrated with the patient medical record system (e.g. so that the CVD event risk score is entered directly in to the patient’s medical record)	51.3	0.770
*2	Is CVD risk advice (e.g. about modifiable risk factors such as diet and exercise) integrated with the patient medical record system?	51.7	0.706
3	Did nurses take part in local/community campaigns or actions on CVD risk prevention (e.g. stop smoking campaigns, fun-runs etc.)?	27.0	0.654
4	Does the practice use a system for recalling populations at risk for preventive care regarding cardio- vascular diseases?	52.1	0.601
5	Is there in general a record in the electronic or paper based patient record that the CVD standardized risk assessment tool has been offered?	48.7	0.577
6	Do you offer regularly two or many consultations to provide advice on patients’ life style?	58.1	0.564
*7	Does the practice use case finding methods to detect patients with cardiovascular risk factors	58.4	0.559
8	Does the practice participate in public health care programmes on life style (physical exercise, stop smoking)?	59.2	0.553
9	Does the practice use a CVD standardized risk assessment tool?	75.7	0.549
*10	Does the practice have an up-to-date directory of prevention activities/organizations available locally (e.g. gyms, walking groups, weight-watchers etc.)?	47.6	0.514
*11	Did all nurses attend ≥ one training/continuing medical education event on CVD within the last 5 years?	52.1	0.488
12	Did your practice participate in a project concerning cardiovascular risk management the last 2 years?	35.2	0.445
*13	Does the practice have a procedure for smoking cessation (e.g. Minimal Intervention Strategy)?	71.9	0.410
**Electronic medical record (EMR) -5 Items**			
1	Does the practice use a computer-supported patient file system?	74.9	0.831
2	Is the computer used for creating medication prescriptions?	79.4	0.809
3	Do the practice doctors have direct access to medical guidelines (either on paper or electronic) in their treatment rooms?	92.1	0.666
4	Does the practice have a procedure for the management of patient information in relation to the review of detailed examination results by the doctor (in terms of outgoing needs)?	76.4	0.633
5	Does the practice have a procedure for the management of patient information in relation to detailed examination results and the documentation of measures that were taken (e.g., blood examinations)?	82.8	0.610
**Chronic care management (CCM) - 6 Items**			
1	Does the practice use a system for recalling patients with diabetes?	58.1	0.752
2	Does the practice use a system for recalling patients with cardio vascular diseases?	53.2	0.705
3	Does the practice use a system for recalling patients with hypertension?	48.3	0.642
4	Did the practice participate in cardiovascular quality improvement projects?	56.9	0.504
5	Does the practice use a system for recalling populations at risk for preventive care regarding influenza?	54.3	0.499
6	Did the practice have a team meeting about quality improvement relating to CVD at least once in the last 15 months?	50.6	0.480
**Quality management (QM) - 3 Items**			
1	Does the practice produce a quality report?	34.5	0.711
2	Has the practice undertaken at least one clinical audit in the last 12 months?	53.2	0.703
3	Does the practice have a critical incident register?	37.8	0.569

* Items that are separately significantly associated with composite quality score.

### Cardiovascular risk management (CVRM) - performance

The composite quality score across eleven quality indicators was 55.9% (Table 4). Composite scores were highest in Slovenia (64.1%) and lowest in the Netherlands (44.3%) (Table 5). The results of single indicator achievement are provided in Table 4.

**Table 4:** Quality indicators included in the composite outcome measure (N = 4223)

**Quality indicators**		**Achieved%**
1 Record of smoking status in the last 15 months (process indicator)		78.3
2 Record of physical activity in the last 15 months (process indicator)		46.2
3 Record of Body Mass Index (BMI) or weight in the last 15 months (process indicator)		67.0
4 Record of blood pressure in the last 15 months (process indicator)		91.6
5 Record of cholesterol in the last 15 months (process indicator)		81.3
6 Record of blood glucose (random or fasting) in the last 15 months (process indicator)		73.7
7 Advice for smokers to quit smoking in the last 15 months (process indicator)		68.1
8 Advice for regular physical activity in the last 15 months (process indicator)		38.6
9 Advice for healthy diet in the last 15 months (process indicator)		46.1
10 Control of blood pressure level (mean blood pressure level of maximal 3 measures ≤ 140/90) (intermediate outcome indicator)		46.7
11 Control of total cholesterol level (≤ 5 mmol/l) (intermediate outcome indicator)		28.4
**Outcome measure: Quality score**		
Percentage of overall achievement across 11 quality indicators (0-100%)		**55.9**

**Table 5:** Practice performance scores on cardiovascular risk management (CVRM) (N = 4,223 individuals; 267 practices)

**Countries**	Composite quality score^*^		
	**Achievement%**	**SD**	Deviance from the overall mean ( *p *)**
Austria	56,5	20,5	⇆
Belgium	59,1	19,0	↑ p = 0.006)
France	62,3	16,5	↑ (p < 0.0001)
Germany	53,5	18,4	↓ (p = 0.006)
Netherlands	44,3	24,5	↓ (p < 0.0001)
Slovenia	64,1	20,8	↑ (p < 0.0001)
Spain	51,8	25,0	↓ (p < 0.0001)
Switzerland	48,4	20,8	↓ (p < 0.0001)
UK	58,4	21,5	↑ (p = 0.006)
Total	55,9	22,2	

⇆ Country score shows no deviance from the mean score of all countries.

↑ Country score higher than the mean score of all countries.

↓ Country score lower than the mean score of all countries.

* Composite quality score = Percentage of overall achievement across 11 quality indicators.

** p-values calculated by t-test.

The 3-level linear regression analysis (Table 6) was based on 3,700 patients (level 1) nested within 240 practices (level 2) and these nested within nine countries (level 3). There were up to 72 patients within each practice and up to 36 practices within each country. There were no differences according to patient and practice characteristics between included patients and those excluded due to missing data. The estimates (regression coefficients, r) indicate the amount of composite quality-indicator scores changes (in%) with each increasing unit of continuous variables (e.g. per 5 year increase for age) or in comparison to a reference category for categorical variables (e.g. ‘female’ versus male). Each regression coefficient is thereby adjusted for all remaining variables included in the model and clustering effects (patients nested in practices and these in countries). At the patient level, neither gender nor age was associated with the performance on CVRM. At the practice level, practice size indicated by full time equivalents (FTE) of GPs and the practice location had no impact on performance. The only significant association (*r* = 1.92; p = 0.0058) was found for the practice score ‘preventive services’ (Table 6) reflecting specific structural and organisational processes of CVD-preventive care (Table 3). The estimate indicates that the composite quality- indicator score will increase by 1.92% with each additional ‘yes-answer’ of the ‘preventive-services’ items (1.92 × 13) resulting in a maximum performance increase of 25%. This maximum difference will be achieved between practices reporting to have none of these ‘preventive-services specific items implemented and those reporting the presence of all 13 items included in the score. The other practice scores had no impact on the quality of CVRM (Table 3 and Table 6).

**Table 6:** Association of explanatory variables with the composite quality score (N = 3,700 individuals within 240 general practices within 9 countries)

	**Estimate**	**[95% CI]**	***P *value**
**Individual level**			
Age (per 5-year)	−0.53	[−1.43; 0.37]	0.2503
Gender: female	−0.99	[−2.33; 0.33]	0.1408
**Practice level**			
FTE GP ≥ 2^1^	2.07	[−1.96; 6.10]	0.3134
Practice location: urban^2^	0.45	[−3.99; 4.89]	0.8429
Practice score ‘PrevServ’^3^	1.92	[0.56; 3.38]	0.0058
Practice score ‘EMR’^4^	0.06	[−2.34; 2.46]	0.9593
Practice score ‘CCM’^5^	−0.88	[−2.22; 0.46]	0.2001
Practice score ‘QM’^6^	−0.21	[−2.11; 1.68]	0.8245

^1^ FTE = Full time equivalent.

^2^ Urban: Practice location in town > 100,000 inhabitants (> 30,000 in Slovenia).

^3^ PrevServ = Preventive Services (score: % of ‘yes answers’ from 13 items) (Table 3).

^4^ EMR = Electronic medical record (score: % of ‘yes answers’ from 5 items) (Table 3).

^5^ CCM = Chronic Care Management (score: % of ‘yes answers’ from 6 items) (Table 3).

^6^ QM = Quality Management (score: % of ‘yes answers’ from 3 items) (Table 3).

The analysis of the 13 single items included in the ‘preventive services’ score showed that 5 of these items were positively associated with the CVRM-performance if included separately in the sensitivity analysis (M4). These five practice capabilities included: the ‘PrevServ’-item 2 (Table 3) - integration of lifestyle advice in the electronic medical record (r = 8.33; p = 0.0006), the ‘PrevServ’-item 7 (Table 3) - use of case-finding methods to identify patients at risk (r = 6.74; p < 0.0001), the ‘PrevServ’-item 10 (Table 3) - the availability of a register of preventive prospects provided by local organisations (r = 4.79; p = 0.0009), the ‘PrevServ’-item 11 (Table 3) - continuous medical education for nurses (r = 6.40; p < 0.0001) and the ‘PrevServ’-item 13 (Table 3) - implementation of a minimal intervention strategy for stop smoking advice (r = 3.78; p = 0.0322). A score of these 5 items (PrevServ_5) resulted in a positive association with the CVRM performance (r = 4.28; p < 0.0001) (Table 7).

**Table 7:** Association of explanatory variables with the composite quality score (N = 3,700 individuals within 240 general practices within 9 countries)

	**Estimate**	**[95% CI]**	***P *value**
**Individual level**			
Age (per 5-year)	−0.52	[−1.42; 0.38]	0.2567
Gender: female	−0.99	[−2.31; 0.35]	0.1488
**Practice level**			
FTE GP ≥ 2^1^	1.60	[−2.12; 5.33]	0.3983
Practice location: urban^2^	0.13	[−3.36; 3.62]	0.9421
Practice score ‘PrevServ_5^3^	4.28	[2.59; 5.97]	<0.0001

^1^ FTE = Full time equivalent.

^2^ Urban: Practice location in town > 100,000 inhabitants (> 30,000 in Slovenia).

^3^ PrevServ_5 = Score of the number of ‘yes answers’ from the following items (Table 3):

Item 2: Is CVD risk advice (e.g. about modifiable risk factors such as diet and exercise) integrated with the patient medical record system?

Item 7: Does the practice use case finding methods to detect patients with cardiovascular risk factors?

Item 10: Does the practice have an up-to-date directory of prevention activities/organizations available locally (e.g. gyms, walking groups, weight-watchers etc.)?

Item 11: Did all nurses attend ≥ one training/continuing medical education event on CVD within the last 5 years?

Item 13: Does the practice have a procedure for smoking cessation (e.g. Minimal Intervention Strategy)?

The random part results of the null model (M0) indicate that the proportion of variance was greater at the practice level ICC = 27.4% compared to the country level ICC = 7.1%. The best model fit was achieved by the last regression model (Table 7). The variables included explained 21.1% of the variation between practices.

## Discussion

We investigated the association between structural and organisational practice characteristics and the quality of cardiovascular risk management (CVRM) provided to patients at high risk for developing CVD. Our study has two main findings: Firstly, performance across the composite quality measure of eleven indicators of CVRM revealed an achievement of only 55.9%. The composite score included indicators of recommended preventive procedures related to the recording of clinical and behavioural risk factors and behavioural advice and also outcomes, i.e. the control of clinical risk factors (blood pressure and cholesterol). Secondly, we found a positive association between CVD-prevention related organisational and functional capabilities of primary care practices and the quality of CVRM provided to patients at high risk of developing CVD in routine primary care settings across different health care systems in Europe. As differences between primary care practices were larger within rather than between countries, these findings suggest that practice characteristics do explicitly have impact on the quality of cardiovascular risk management in primary care.

Composite measures of quality indicators were used in previous studies to assess quality of care for multiple conditions in primary care [19,20]. The result of the composite measure for recommended chronic care (56.1%) before implementation of quality improvement approaches such as the QOF was quite similar to the composite quality measure found in our study [19,21].

There are many ways to describe practice characteristics and their linkage to quality of care in various settings resulting in heterogeneous findings [11-13,22-27].

In our study, we had included practices from different health care systems with a wide variation concerning the use of information technology and the implementation of recommended procedures of CVRM. We described practice capabilities using four scores of practice characteristics related to generic quality-management aspects, chronic care management, the use of EMR-systems and specific CVD-preventive care features, items of which were derived from EPA instruments [14,15,17].

Only one score, the preventive-services score that contained CVD-prevention related organisational and functional practice features in a more specific way, was significantly associated with clinical performance on CVRM. Within these characteristics (Table 3) five practice characteristics were sparately associated with higher quality of CVRM. These characteristics were related to the integration of lifestyle advice procedures in the electronic medical record system, the implementation of case-finding methods to identify patients at risk, the provision of a register of preventive prospects of local organisations, continuous medical education for nurses and the implementation of a minimal intervention strategy for stop smoking advice.

Most previous studies investigating the relationship between practice characteristics and quality of health care have focused on the delivery of chronic care rather than on prevention. The Chronic Care Model (CCM) is a widely adopted framework to enhance evidence based chronic care in primary care [28] and elements of the CCM have been associated with improved quality of care and patient outcomes [22,29,30]. A study in two U.S. managed care organisations found that only few practice characteristics were related to the quality of care such as multi-site practices and those with an intermediate number of member physicians [11]. Other characteristics such as financial incentives or the use of EHR without determining functional capacities had no effect on quality measures [11].

Holmboe et al. used a measurement tool that assessed the presence of practice structure and care process elements to qualify primary care practices as ‘medical homes’, a widely accepted framework of patient-centred care [13]*.* They did not find an association between ‘successful’ practice systems according to these measurements and the quality of care for multiple diseases and conditions [13]. It is assumed that practices in study settings such as managed care organisations with high quality standards may not significantly differ from each other [11]. This may explain why we found associations of practice characteristics and quality of care in a European sample with large variation between practices, whereas another study in practices that were granted at the highest attainable level of medical home recognition by the National Committee for Quality Assurance (NCQA) found no relationship between systems and performance measures [31]. The CCM-score in our study was not associated with the quality outcome. A possible reason therefore may be that focussing on chronic care management does not automatically imply the delivery of high quality preventive services for patients at risk without an established chronic disease [32]. In fact, promoting the delivery chronic care management by providing financial incentives may rather lead to the disregard of other patient groups and services [33].

Other studies with a broader focus on health care including preventive services have found only few relationships between practice characteristics and clinical performance measures [12,13,34]: It has been argued that general quality assurance processes implemented in practices such as the regular collecting of data for audit purposes have no impact on quality of care [12]. This result is comparable to our study were the QM-score was not associated with better outcomes. In line with our study results, the presence of an electronic medical record system (EMR) was not associated with quality of care but rather the availability and the use of specific EHR-features [13,34]. It was reported that the assessment of practice characteristics must consider more than practice structure, even the practice staff working in the practice system, their patients and the crucial interactions among the system, physicians, patients and community resources [13].

It is notable that the five practice capabilities associated with a higher quality of CVRM in our study were mainly related to the identification of patients at risk and lifestyle management procedures. The identification of patients at risk and lifestyle management are key components of cardiovascular prevention [35], time consuming and generally not reimbursed in primary care [36]. The delivery of effective preventive care services may therefore require more support from health care systems [36,37].

### Strengths and limitations

We used multilevel modelling to identify predictors of the quality of CVRM in one model that adjusted for the hierarchical structure of our data and for the remaining variables included in the model. Hierarchical models combine information across units to produce accurate and well calibrated prediction of outcomes [38]. This analytical approach can also be used to describe the amount of variation at each level that makes it possible to separate practice variation from country (health system) influence. We used a medical record audit to measure performance on CVRM that provides valid data but is also dependent on the quality of documentation. The practice measures were self-reported by physicians, however, it has been demonstrated that lead physicians tend to underestimate the features they have in their practices [39].

In some countries it was difficult to enrol 36 practices, as intended in the power analysis, and that may affect the generalisabilty of our results. The patient sample included a greater proportion of men due to the inclusion criterion ‘men over 60 years’ as one of four risk factors (Table 1) that were used for inclusion. However, the results of the multilevel analyses were controlled for gender influences. The multilevel analyses led to a decrease of the total number of cases due to missing data, as we conducted a complete cases analysis. However, we made sure that both samples did not differ between each other according to patient and practice characteristics.

The cross-sectional design of our study does not allow causal attribution of performance to structural and organisational practice characteristics. However, because of the international character of the study, a larger external validity can be expected.

## Conclusions

Our study results suggest that the quality of cardiovascular risk management in individuals at high risk for developing CVD is improvable with mean indicator achievement of only 55,9% and with a greater variation in practice scores within rather than between countries. Quality improvement is associated with practice capabilities related to the identification of patients at risk (risk assessment), continuous education for nurses and lifestyle management procedures integrated in to the electronic medical record system. The implementation of these preventive services in primary routine care may require health care system support.

## Competing interests

The authors declare that they have no competing interests.

## Authors’ contributions

MW developed the overall outline of the EPA Cardio project. SC coordinated the international selection of performance indicators, on which the EPA Cardio audit instrument is based. SL coordinated the development and pilot-testing of the measurements and wrote the draft and the final version of this paper. JvL contributed to the development and selection of measurements. JR supervised statistical analyses. JS and DO were supervisors of the research reported in this paper. All authors critically assessed and approved this paper. All authors read and approved the final manuscript.
